# The mitochondrial Ras-related GTPase Miro: views from inside and outside the metazoan kingdom

**DOI:** 10.3389/fpls.2014.00350

**Published:** 2014-07-16

**Authors:** Shohei Yamaoka, Ikuko Hara-Nishimura

**Affiliations:** ^1^Graduate School of Biostudies, Kyoto UniversityKyoto, Japan; ^2^Graduate School of Science, Kyoto UniversityKyoto, Japan

**Keywords:** mitochondria, Miro, Ras GTPase, metazoan, *Saccharomyces cerevisiae*, *Dictyostelium discoideum*, *Arabidopsis thaliana*

## Abstract

Miro GTPase, a member of the Ras superfamily, consists of two GTPase domains flanking a pair of EF hand motifs and a C-terminal transmembrane domain that anchors the protein to the mitochondrial outer membrane. Since the identification of Miro in humans, a series of studies in metazoans, including mammals and fruit flies, have shown that Miro plays a role in the calcium-dependent regulation of mitochondrial transport along microtubules. However, in non-metazoans, including yeasts, slime molds, and plants, Miro is primarily involved in the maintenance of mitochondrial morphology and homeostasis. Given the high level of conservation of Miro in eukaryotes and the variation in the molecular mechanisms of mitochondrial transport between eukaryotic lineages, Miro may have a common ancestral function in mitochondria, and its roles in the regulation of mitochondrial transport may have been acquired specifically by metazoans after the evolutionary divergence of eukaryotes.

## INTRODUCTION

Mitochondria are essential organelles for aerobic energy production and metabolism in eukaryotic cells. They frequently undergo changes in morphology and intracellular distribution through fusion, fission, and cytoskeleton-dependent transport, presumably to sustain their functional homeostasis. Severely damaged mitochondria can be the target of an autophagic degradation mechanism termed mitophagy (Logan, 2010; Westermann, 2010; Chan, 2012; Otera et al., 2013; Friedman and Nunnari, 2014). The functions and dynamics of mitochondria are linked to evolutionarily conserved proteins localized to the mitochondrial outer membrane. For example, voltage-gated anion channels (VDAC) regulate the flow of metabolites, including ATP and ADP, across the outer membrane (Lemasters and Holmuhamedov, 2006; Colombini, 2012). The translocase of the outer mitochondrial membrane (TOM) complex is the main pathway for mitochondrial protein transport, while the topogenesis of mitochondrial outer membrane β-barrel (TOB)/sorting and assembly machinery (SAM) complex plays an important role in the assembly of outer membrane proteins (Pfanner et al., 2004; Neupert and Herrmann, 2007; Endo and Yamano, 2010). Dynamin-related GTPases are recruited to the outer membrane and form a ring-like oligomer that constricts mitochondria, leading to fission (Kuroiwa et al., 2006; Bui and Shaw, 2013; Chappie and Dyda, 2013).

The Miro protein is a mitochondrial outer membrane-localized GTPase that is highly conserved throughout eukaryotes. In metazoans, Miro is a component of the protein complex that regulates mitochondrial transport. However, accumulating evidence from studies of non-metazoans, including plants, suggests that Miro is involved in the maintenance of mitochondrial morphology and homeostasis. Here, we review the studies investigating Miro GTPases in diverse eukaryotes and reconsider the molecular functions and physiological roles of Miro in the light of eukaryotic evolution.

## MOLECULAR STRUCTURE OF Miro GTPases

**FIGURE 1:**
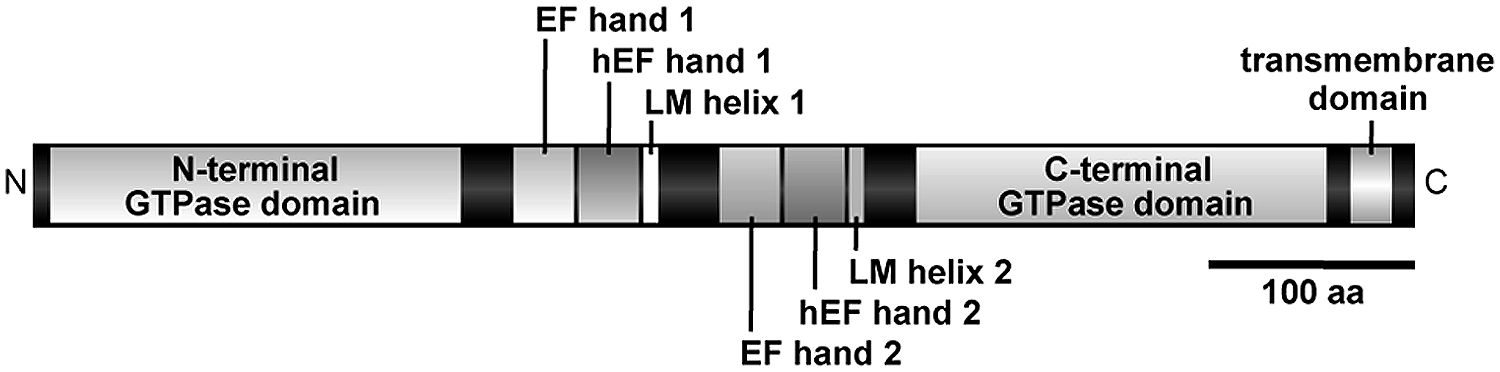
**Molecular structure of the Miro GTPase.** Schematic representation of the molecular structure of Miro according to Klosowiak et al. (2013). Domain names are described in the text. The bar indicates a length corresponding to 100 amino acid residues.

Miro GTPase is anchored to the mitochondrial outer membrane by its C-terminal transmembrane domain, leaving its N-terminus exposed to the cytoplasm. Its cytoplasmic region contains two structurally distinct GTPase domains that are separated by a pair of EF hand motifs (EF hands 1 and 2; Fransson et al., 2003, 2006; Frederick et al., 2004; Guo et al., 2005; Yamaoka and Leaver, 2008; Vlahou et al., 2011; **Figure 1**). Miro was originally classified as an atypical Rho GTPase based on sequence similarity of the N-terminal GTPase domain to Rho family proteins (Fransson et al., 2003). However, later studies found that both GTP domains lack the conserved G-3 DxxG motif (Bourne et al., 1991) and the Rho-specific insert region (Freeman et al., 1996; Walker and Brown, 2002), suggesting that they represent two independent subfamilies of the Ras GTPase superfamily (Frederick et al., 2004; Wennerberg and Der, 2004; Boureux et al., 2007; Reis et al., 2009). A recent study of Miro in fruit flies showed that its C-terminal GTPase domain is most structurally similar to Rheb, a Ras subfamily member (Mazhab-Jafari et al., 2012; Klosowiak et al., 2013). Correspondingly, the catalytic rates of the two GTPase domains of the budding yeast Miro homolog Gem1p are comparable to those of the Ras family and are significantly slower than those of the dynamin family (Koshiba et al., 2011). The two conserved EF hands of Miro have been shown to bind Ca^2+^ (MacAskill et al., 2009; Koshiba et al., 2011) and the flanking regions of the EF hands are highly conserved among eukaryotes (Vlahou et al., 2011). Klosowiak et al. (2013) showed that these regions contain non-canonical “hidden” EF hands (hEF hands 1 and 2) followed by single helices (LM helices 1 and 2; **Figure 1**). The hEF hands have a typical helix–loop–helix structure and stabilize the adjacent EF hands by forming an anti-parallel EF hand β-scaffold. The structure of the Miro LM helices resembles extrinsic ligands bound to EF hand proteins, as reported for the protein complexes of Troponin I and Troponin C (Vinogradova et al., 2005) and molluscan myosin heavy chain and light chain (Houdusse and Cohen, 1996; Klosowiak et al., 2013). The EF–hEF hand pair combined with the LM helix can be found in various Ca^2+^-binding proteins including the pollen protein polcalcin (Neudecker et al., 2004), the retinal protein recoverin (Tanaka et al., 1995; Ames et al., 2006), and human guanylate cyclase-activating protein GCAP3 (Stephen et al., 2006). Miro is a monomeric protein that assumes a compact and linear conformation in solution and undergoes no significant conformational rearrangement into another stable form and/or oligomerization in response to ions or nucleotides (Klosowiak et al., 2013). Conformational changes of Miro may require an interacting partner, similar to other EF hand proteins (Grabarek, 2006; Klosowiak et al., 2013).

## Miro GTPases EMERGED BEFORE THE DIVERGENCE OF EUKARYOTES

An extensive phylogenetic analysis by Vlahou et al. (2011) showed that at least one Miro homolog is present in almost all eukaryotic genomes. The phylogeny of Miro homologs shows a clear correlation with that of eukaryotic species and no obvious homolog can be found in prokaryotes. This suggests that Miro appeared at an early stage of eukaryotic evolution, perhaps before the divergence of extant eukaryotic species (Vlahou et al., 2011); however, the exceptions are found in several species. First, Miro is absent from eukaryotic species that possess mitosomes and hydrogenosomes instead of canonical aerobic mitochondria, including the phylum Microsporidia and the genus *Entamoeba*. Second, the genomes of several species possessing aerobic mitochondria, such as the phylum Apicomplexa and the order Mamiellales, lack Miro. Third, Miro homologs in the order Trypanosomatid have a non-functional version of EF hand 2 and lack the N-terminal GTPase domain, possessing instead a novel domain without similarity to any other defined sequences. Fourth, in the class Oligohymenophorea, the C-terminal Miro GTPase domains are replaced by sequences that are not conserved, even within the class. Fifth, Miro homologs from Amoebozoa and Stramenopiles have a non-functional C-terminal GTPase domain that lacks the conserved residues. These variations are found separately in different eukaryotic lineages, suggesting that the molecular structure of Miro was modified independently to meet the functional demands of the protein in each lineage after the divergence of eukaryotes (Vlahou et al., 2011).

## METAZOAN Miro GTPases

### Miro IS A Ca^2+^-DEPENDENT REGULATOR OF MITOCHONDRIAL TRANSPORT IN METAZOANS

Mitochondrial transport is essential for neuronal energy supply to the axons and for the transmission of signals from the cell body to the synaptic junctions. Disruption of mitochondrial distribution in neurons is deleterious and is associated with neurodegenerative diseases, including dominant optic atrophy, Charcot-Marie-Tooth, Alzheimer’s, Huntington’s, and Parkinson’s diseases (Chen and Chan, 2009; Harris et al., 2012; Saxton and Hollenbeck, 2012). In axons, mitochondria are transported along microtubules by the action of kinesins and dyneins as anterograde and retrograde motors, respectively. A screening of genetically mosaic mutant fruit flies identified allelic lethal mutations of Miro that cause abnormal larval locomotion and premature lethality. In the mutant neurons, mitochondria are abnormally clustered in the cell body and are often absent from the synaptic terminals, suggesting a requirement for Miro in anterograde mitochondrial transport along axons (Guo et al., 2005). Subsequent studies showed that Miro forms a protein complex with the kinesin-associated protein Milton (Stowers et al., 2002), which recruits kinesins to mitochondria for anterograde transport (Glater et al., 2006). Two mammalian Milton homologs, GRIF-1 (also known as OIP98, huMilt2, or TRAK2) and OIP106 (also known as huMilt1 or TRAK1; Beck et al., 2002; Stowers et al., 2002; Iyer et al., 2003; Brickley et al., 2005; Smith et al., 2006), associate with Miro, suggesting that Miro is a component of a conserved protein complex involved in mitochondrial transport (Fransson et al., 2006; Wang and Schwarz, 2009; Weihofen et al., 2009). Mitochondrial transport is dependent on cytosolic Ca^2+^ (Rintoul et al., 2003; Yi et al., 2004), and a role for Miro in its regulation has been demonstrated (Saotome et al., 2008; MacAskill et al., 2009; Wang and Schwarz, 2009; Chang et al., 2011). However, several different models for the underlying mechanism have been proposed. Wang and Schwarz (2009) proposed that Miro interacts with kinesin via Milton independently of Ca^2+^. In this model, increased cytosolic Ca^2+^ causes the N-terminal kinesin motor domain to dissociate from microtubules and interact with Miro, resulting in the arrest of mitochondrial transport (Wang and Schwarz, 2009). MacAskill et al. (2009) proposed an alternative model by which Miro directly associates with kinesin without the aid of Milton. In this model, an increase in cytosolic Ca^2+^ inhibits the association and allows Miro to be released from kinesin (MacAskill et al., 2009). Accumulating evidence suggests that Miro is also involved in the regulation of retrograde mitochondrial transport (Russo et al., 2009; Wang and Schwarz, 2009; Misko et al., 2010; Morlino et al., 2014).

Several neuron-specific proteins that modify the function of Miro in mitochondrial transport were identified recently. Syntaphilin associates with the kinesin that is released from Ca^2+^-binding Miro, leading to stationary mitochondrial docking through interaction with microtubules in axons (Chen and Sheng, 2013). The hypoxia-inducible protein HUMMR interacts with Miro and the mammalian Milton homologs, and biases axonal transport of mitochondria in the anterograde direction, presumably for the maintenance of neuronal functions and survival during hypoxia (Li et al., 2009). Alex3, another protein associated with Miro-mediated mitochondrial transport machinery in neurons, is unique to Eutherian mammals. Alex3 originated through a Eutherian-specific gene duplication and may be linked to the increase in brain complexity in Eutherians (López-Doménech et al., 2012).

### Miro IS A TARGET OF PARKIN-MEDIATED DEGRADATION IN MAMMALIAN CELLS

Recent evidence suggests that Miro-mediated mitochondrial transport is associated with Parkinson’s disease (PD), a common neurodegenerative disorder characterized by motor disturbances. A form of autosomal recessive juvenile PD is caused by mutations in the mitochondria-targeted Ser/Thr kinase PINK1 and the E3 ubiquitin ligase Parkin. PINK1 and Parkin operate together in a common pathway involved in the regulation of multiple aspects of mitochondrial quality control, including mitochondrial biogenesis, fusion and fission, transport, and mitophagy (Chen and Chan, 2009; Scarffe et al., 2014). PINK1 and Parkin are recruited to the damaged mitochondrial outer membrane, where they phosphorylate and ubiquitinate various proteins including VDACs and the mitochondrial fusion proteins mitofusins (Geisler et al., 2010; Ziviani et al., 2010; Chen and Dorn, 2013). Recent studies showed that Miro is also a target of the PINK1-Parkin pathway, although its ubiquitination pattern remains unclear (Weihofen et al., 2009; Wang et al., 2011; Liu et al., 2012; Sarraf et al., 2013; Birsa et al., 2014). The Parkin-mediated proteasomal degradation of Miro leads to the dissociation of kinesin from mitochondria and the subsequent arrest of mitochondrial transport. These events may quarantine the damaged mitochondria to facilitate mitophagic clearance (Wang et al., 2011; Liu et al., 2012; Birsa et al., 2014).

### Miro IS INVOLVED IN MITOCHONDRIAL MORPHOLOGY AND Ca^2+^ HOMEOSTASIS IN METAZOANS

Several studies suggest that metazoan Miro plays different roles in mitochondrial dynamics and function other than mitochondrial transport. Overexpression of Miro and its mutant proteins influences mitochondrial morphology (Fransson et al., 2003, 2006; Glater et al., 2006; Saotome et al., 2008; Weihofen et al., 2009). Overexpression experiments showed that Miro and Drp1, a dynamin GTPase associated with mitochondrial fission, function in an antagonistic manner in mitochondrial morphology, suggesting that Miro may play a role in the maintenance of mitochondrial morphology by suppressing Drp1-mediated mitochondrial fission (Saotome et al., 2008). Miro is also likely to be involved in mitochondrial Ca^2+^ homeostasis. Chang et al. (2011) showed that mitochondrial Ca^2+^ content is negatively correlated with the velocity of mitochondrial transport. Overexpression of a non-functional EF hand mutant version of Miro decreased Ca^2+^ entry into mitochondria, suggesting that Miro is primarily involved in the regulation of mitochondrial Ca^2+^ influx and homeostasis, which, in turn, influences mitochondrial transport (Chang et al., 2011; Niescier et al., 2013).

## NON-METAZOAN Miro GTPases

### Miro IS INVOLVED IN THE MAINTENANCE OF MITOCHONDRIAL MORPHOLOGY AND INHERITANCE IN *Saccharomyces cerevisiae*

In the budding yeast *Saccharomyces cerevisiae*, the single-copy Miro homolog Gem1p plays a role in mitochondrial morphology and inheritance. The mitochondrial compartment in wild-type yeast is characterized by a branched network of tubular structures at the cell cortex (Koning et al., 1993; Frederick et al., 2004). In the *gem1* knockout mutant, mitochondria show a globular, collapsed tubular, or grape-like morphology without an obvious impact on the mitochondrial membrane structures, suggesting that Gem1p is required for the maintenance of mitochondrial morphology. Amino acid substitution experiments suggest that the function of Gem1p in the regulation of mitochondrial morphology requires both the GTPase domains and the EF hands (Frederick et al., 2004). The *gem1* knockout mutant also shows impaired cell growth on synthetic glycerol media, implying that Gem1p is required for proper mitochondrial respiration (Frederick et al., 2004). Genetic analysis showed that the *GEM1* pathway is independent from the known mitochondrial morphology pathways, including those related to mitochondrial fusion and fission (Frederick et al., 2004). Further analyses suggested that Gem1p is involved in a pathway that influences mitochondrial inheritance and is independent of other pathways mediated by the myosin-interacting proteins Mmr1p and Ypt11p (Frederick et al., 2004, 2008).

### Miro PLAYS A ROLE IN MITOCHONDRIA–ENDOPLASMIC RETICULUM INTERACTION

Accumulating evidence suggests that mitochondria and the endoplasmic reticulum (ER) physically interact with one another and play roles in various cellular processes, including phospholipid biosynthesis and mitochondrial fission (Rowland and Voeltz, 2012; Friedman and Nunnari, 2014; Vance, 2014). Kornmann et al. (2009) showed that loss of MDM12, a subunit of the ER–mitochondria encounter structure complex (ERMES) that is essential for various mitochondrial functions (Boldogh et al., 2003; Youngman et al., 2004; Meisinger et al., 2007), can be rescued by an artificial tethering of mitochondria and the ER. ERMES localizes to mitochondria–ER contact sites and is visualized as punctate structures, suggesting its critical role in mitochondria–ER interactions (Kornmann et al., 2009). Gem1p interacts with ERMES; however, this interaction is not required for the assembly of the ERMES complex (Kornmann et al., 2011; Stroud et al., 2011). Imaging analysis suggests that Gem1p negatively regulates ER-associated mitochondrial fission (Murley et al., 2013). Studies suggest that the mitochondria–ER interaction mediates the exchange of phosphatidylserine (PS) and phosphatidylethanolamine (PE) between the two organelles, allowing phosphatidylcholine (PC) biosynthesis (Rowland and Voeltz, 2012; Vance, 2014). Disruption of ERMES impairs the conversion of PS to PC, and knockout of *gem1* has deleterious effects in mutants defective in PS synthesis, suggesting that Gem1p plays a role in lipid exchange through the activity of ERMES (Kornmann et al., 2009, 2011). However, several discrepancies remain to be clarified (Nguyen et al., 2012; Vance, 2014).

### Miro IS INVOLVED IN MITOCHONDRIAL HOMEOSTASIS IN *Dictyostelium discoideum*

The slime mold *Dictyostelium discoideum* has a single copy of the *gemA* gene, which encodes a Miro homolog. The *gemA* knockout mutants show impaired cell growth on nutrient media without any obvious defects in cell division, implying that GemA is involved in mitochondrial function (Vlahou et al., 2011). In *D. discoideum*, mitochondrial transport is primarily mediated by microtubules (Fields et al., 2002; Vlahou et al., 2011). The *gemA* mutants show no obvious phenotype with respect to mitochondrial size, morphology, or intracellular distribution. Co-immunoprecipitation assays suggest that GemA does not associate with the *Dictyostelium* kinesin Kif5. These findings indicate that Miro does not play a role in microtubule-dependent mitochondrial transport in *D. discoideum* (Vlahou et al., 2011). However, the absence of *gemA* compromises multiple aspects of mitochondrial function including total mitochondrial mass, ATP accumulation, and oxygen consumption, but does not influence glucose consumption, reactive oxygen species (ROS) generation, or mitochondrial membrane potential. This suggests that the primary role of Miro in *D. discoideum* is the regulation of mitochondrial homeostasis rather than mitochondrial transport (Vlahou et al., 2011).

### Miro INFLUENCES MITOCHONDRIAL MORPHOLOGY IN *Arabidopsis thaliana*

Plant mitochondria are uniformly spherical and undergo frequent fusion and fission and actin-dependent transport. The *Arabidopsis thaliana* genome contains three Miro homologs, namely, *MIRO1* (At5g27540), *MIRO2* (At3g63150), and *MIRO3* (At3g05310). *MIRO1* and *MIRO2* are expressed throughout the plant (Yamaoka and Leaver, 2008), whereas *MIRO3* is expressed specifically in the endosperm (Winter et al., 2007; Bassel et al., 2008; Day et al., 2008). Insertional mutation of the *MIRO1* gene has multiple effects on plant growth and development including impairment of pollen tube growth and embryonic lethality at an early stage (Yamaoka and Leaver, 2008; Sørmo et al., 2011). Mutation of the *MIRO2* gene enhances the *miro1* mutant phenotype and includes defects in female gametogenesis associated with delayed polar nuclear fusion (Sørmo et al., 2011). Imaging analyses showed abnormally enlarged mitochondria in the *miro1* mutant, although their inner membrane structures were likely to be normal (Yamaoka and Leaver, 2008). The *miro1* mutation also influences mitochondrial inheritance during cell division at an early stage of embryogenesis (Yamaoka et al., 2011); however, the mutant mitochondria undergo continuous cytoplasmic streaming in an actin-dependent manner. In addition, an obvious Milton homolog is absent from the *Arabidopsis* genome. These findings suggest that the primary role of *Arabidopsis* Miro is in the maintenance of mitochondrial morphology rather than actin-dependent mitochondrial transport (Yamaoka and Leaver, 2008; Yamaoka et al., 2011).

## CONCLUDING REMARKS

Multiple lines of evidence suggest that, in metazoans, Miro is primarily involved in the Ca^2+^-dependent regulation of mitochondrial transport; however, in non-metazoans, Miro plays a primary role in the maintenance of mitochondrial morphology and homeostasis (**Table 1**). The molecular mechanisms of mitochondrial transport differ between eukaryotic lineages. In metazoans, microtubule-dependent mitochondrial transport is well defined, whereas in budding yeast, mitochondrial transport and inheritance are mediated by multiple myosin-dependent and -independent pathways (Boldogh and Pon, 2007; Frederick and Shaw, 2007; Frederick et al., 2008; Förtsch et al., 2011). Plants use actin filaments and myosins for mitochondrial transport (Avisar et al., 2008; Peremyslov et al., 2008; Prokhnevsky et al., 2008; Sparkes et al., 2008; Avisar et al., 2009), although the molecular interactions linking mitochondria and myosins remain elusive. These differences suggest that each of the eukaryotic lineages independently developed their own mitochondrial transport machinery after divergence from the ancestral eukaryotic cell. In contrast, Miro is present in almost all eukaryotes, and the phylogeny of Miro homologs and eukaryotic lineages correspond well, suggesting that Miro emerged before the divergence of eukaryotes. Therefore, it is possible that Miro has a common ancestral function in every eukaryote that is related to the maintenance of mitochondrial morphology and homeostasis, while it acquired a role in the regulation of mitochondrial transport specifically in metazoans. The presence of cell-type-specific and lineage-specific Miro-interacting partners (**Table 1**) implies that the molecular nature of Miro promotes its physical interaction with multiple types of proteins. Identification of Mito-interacting partners in non-metazoans will provide further insights into the functions of Miro and the evolution of mitochondrial functions and dynamics.

**Table 1:** Molecular function and interacting proteins of Miro GTPase from various eukaryotes.

		Gene names	Mitochondrial functions	Interacting proteins
Metazoans	Mammals	Miro-1, Miro-2^1^ (humans)	Microtubule-dependent transport^6-20^	GRIF-1/OIP98/huMilt2/TRAK2^6,9^
				OIP106/huMilt1/TRAK1^6,14^
				Kinesin^9^
				Dynein^20^
				PINK1^14-17^
				Parkin^15-17^
				Mitofusin2^19^
				HUMMR (neuron-specific)^12^
				Alex3 (Eutherian neuron-specific)^13^
			Morphology^1,6,7,14,21^	–
			Ca^2+^ homeostasis^18^	–
			Mitochondria-ER interaction^22^	–
	*Drosophila melanogaster*	*dMiro*^2^/*Miro*^21^	Microtubule-dependent transport^2,21^	Milton^8,21^

Non-metazoans	*Saccharomyces cerevisiae*	*GEM1*^3^	Morphology^3,22,23,26^	–
			Inheritance^3,23^	–
			Mitochondria-ER interaction^22,24,25,26^	Mdm34p^22,25^
				Mmm1p^22,25^
				Mdm10p^25^
				Mdm12p^25^
	*Dictyostelium discoideum*	*gemA*^4^	Homeostasis^4^	–
	*Arabidopsis thaliana*	*MIRO1, MIRO2, MIRO3*^5^	Morphology^5,27^	–
			Inheritance^27^	–

*Summary table showing gene names, mitochondrial functions, and interacting proteins of Miro from mammals, fruit fly (*D. melanogaster*), budding yeast (*S. cerevisiae*), slime mold (*D. discoideum*), and plant (*A. thaliana*). Superscript numbers correspond to the following references: (1) Fransson et al. (2003); (2) Guo et al. (2005); (3) Frederick et al. (2004); (4) Vlahou et al. (2011); (5) Yamaoka and Leaver (2008); (6) Fransson et al. (2006); (7) Saotome et al. (2008); (8) Wang and Schwarz (2009); (9) MacAskill et al. (2009); (10) Russo et al. (2009); (11) Chen and Sheng (2013); (12) Li et al. (2009); (13) López-Doménech et al. (2012); (14) Weihofen et al. (2009); (15) Wang et al. (2011); (16) Liu et al. (2012); (17) Birsa et al. (2014); (18) Chang et al. (2011); (19) Misko et al. (2010); (20) Morlino et al. (2014); (21) Glater et al. (2006); (22) Kornmann et al. (2011); (23) Frederick et al. (2008); (24) Kornmann et al. (2009); (25) Stroud et al. (2011); (26) Murley et al. (2013); (27) Yamaoka et al. (2011).*

## Conflict of Interest Statement

The authors declare that the research was conducted in the absence of any commercial or financial relationships that could be construed as a potential conflict of interest.
